# Case Report of Bone Marrow-Sparing Proton Therapy Craniospinal Irradiation for Central Nervous System Myelomatosis

**DOI:** 10.7759/cureus.1885

**Published:** 2017-11-28

**Authors:** Greg Kauffmann, Robin A Buerki, Rimas V Lukas, Vinai Gondi, Steven J Chmura

**Affiliations:** 1 Department of Radiation and Cellular Oncology, University of Chicago; 2 Neurological Surgery, UCSF School of Medicine; 3 Neuro-Oncology, Northwestern University, Feinberg School of Medicine; Chicago, Illinois; 4 Radiation Oncology, Northwestern Medicine Chicago Proton Center, Warrenville, Il

**Keywords:** multiple myeloma, craniospinal irradiation, proton therapy

## Abstract

Central nervous system (CNS) involvement is rare but it is an increasingly recognized complication of the multiple myeloma. The craniospinal radiotherapy is a standard treatment option, however, it may be challenging to deliver due to hematologic toxicity in the patients with multiple prior systemic therapies. We report a case of CNS myelomatosis in a patient with prior stem cell transplant multiple systemic therapies treated with bone marrow-sparing proton therapy craniospinal irradiation, with the dramatic clinical response and minimal hematologic toxicity.

## Introduction

Central nervous system (CNS) involvement in the multiple myeloma (MM) is rare and typically present in the setting of advanced disease and multiple prior therapies. The incidence may be increasing, possibly related to better detection and improved survival of myeloma patients in the era of novel systemic agents. The treatment options include intrathecal therapy, systemic therapy, and radiation therapy. Unfortunately, the prognosis remains extremely poor despite improvements in the systemic therapies. Herein, we report a case of CNS myelomatosis (CNS MM) with dramatic clinical and radiographic response to bone marrow-sparing proton therapy craniospinal irradiation (CSI).

## Case presentation

A 44-year-old male was diagnosed with International Staging System stage III, lambda light chain multiple myeloma, after presenting with progressive fatigue, hypercalcemia, and renal dysfunction. He received two months of Lenalidomide and Dexamethasone but was temporarily lost to follow-up. Approximately one year after diagnosis, he resumed therapy and completed four cycles of bortezomib, Lenalidomide, and Dexamethasone with the complete response based on normalization of his kappa/lambda ratio and negative bone marrow biopsy. He then underwent an uncomplicated autologous stem cell transplant with melphalan conditioning followed by maintenance Lenalidomide.

Approximately three years and three months after initial diagnosis, he presented with left eye visual changes. The magnetic resonance imaging (MRI) of the orbits revealed contrast-enhancement and widening of the left orbital nerve concerning for malignant infiltration. In addition, two subcentimeter, enhancing parenchymal lesions were noted in the cerebellum. A lumbar puncture confirmed monoclonal plasma cells in the cerebrospinal fluid (CSF). The repeat bone marrow biopsy was negative for myeloma involvement and restaging computerized tomography (CT) scan was unremarkable.

**Figure 1:**
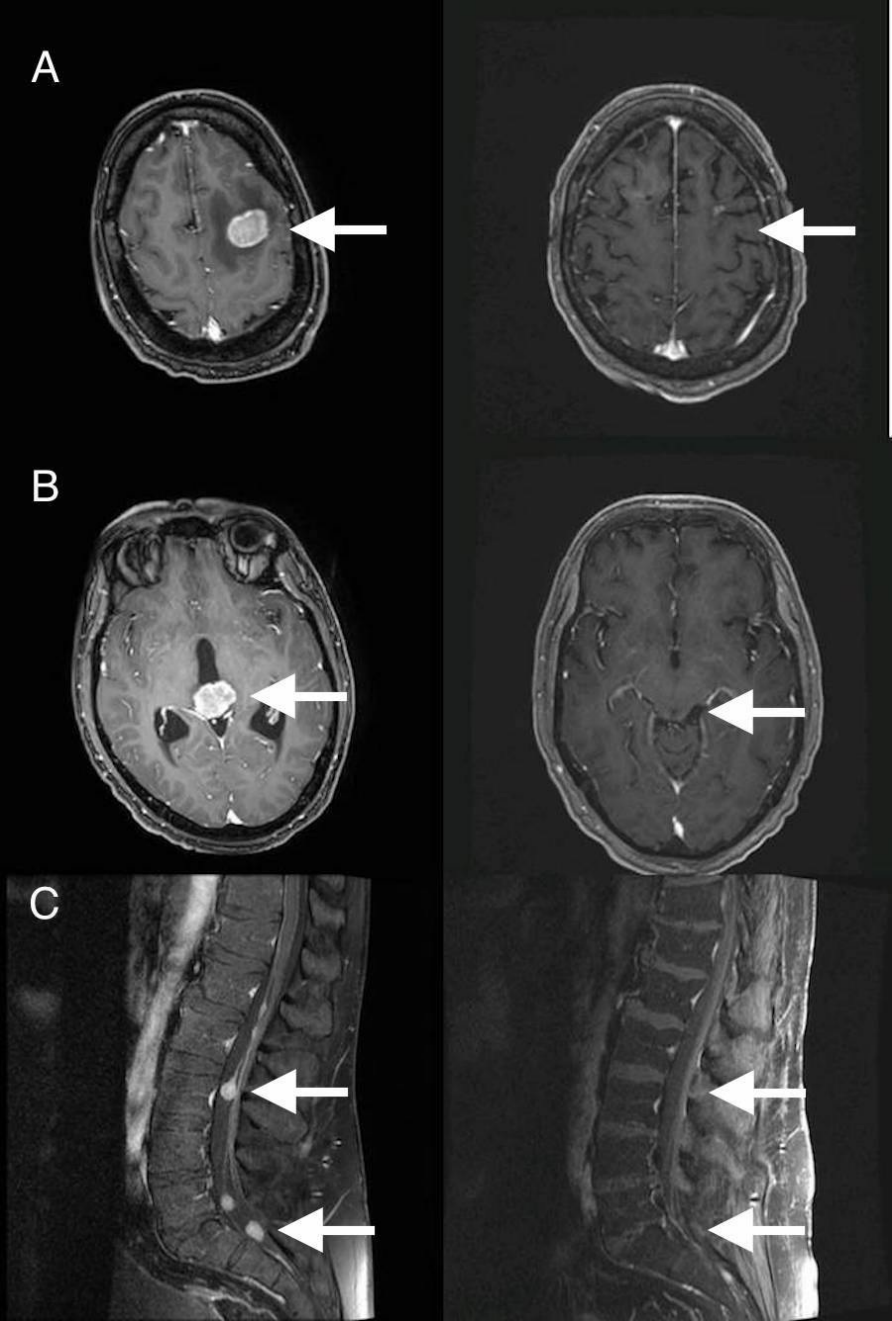
Pre- and post-treatment neuroimaging, nine months after craniospinal irradiation. A) Left frontal intracranial lesion, pre- (left) and post- (right) treatment. B) Pineal region intracranial mass, pre- (left) and post- (right) treatment. C) Multiple enhancing nodules in cauda equina, pre- (left) and post-treatment.

He received three cycles of high dose methotrexate (8 g/m2) followed by leucovorin rescue with persistent plasma cells in the CSF. He subsequently developed a rapid decline in neurologic status, including right-sided hemiparesis, left lower extremity radicular pain, and confusion. The repeat neuro-imaging demonstrated the progression of multiple intracranial lesions and increased leptomeningeal enhancement. Of note, there was a large mass in the left frontal region and another in the pineal region with mass effect on the cerebral aqueduct causing hydrocephalus. Imaging of the complete spine revealed diffuse leptomeningeal disease along the nerve roots of the cauda equina with multiple enhancing nodules (Figure 1). There was no radiographic evidence of osseous involvement throughout the spine. He then underwent endoscopic third ventriculostomy for the relief of obstructive hydrocephalus and was referred for radiotherapy.

**Figure 2:**
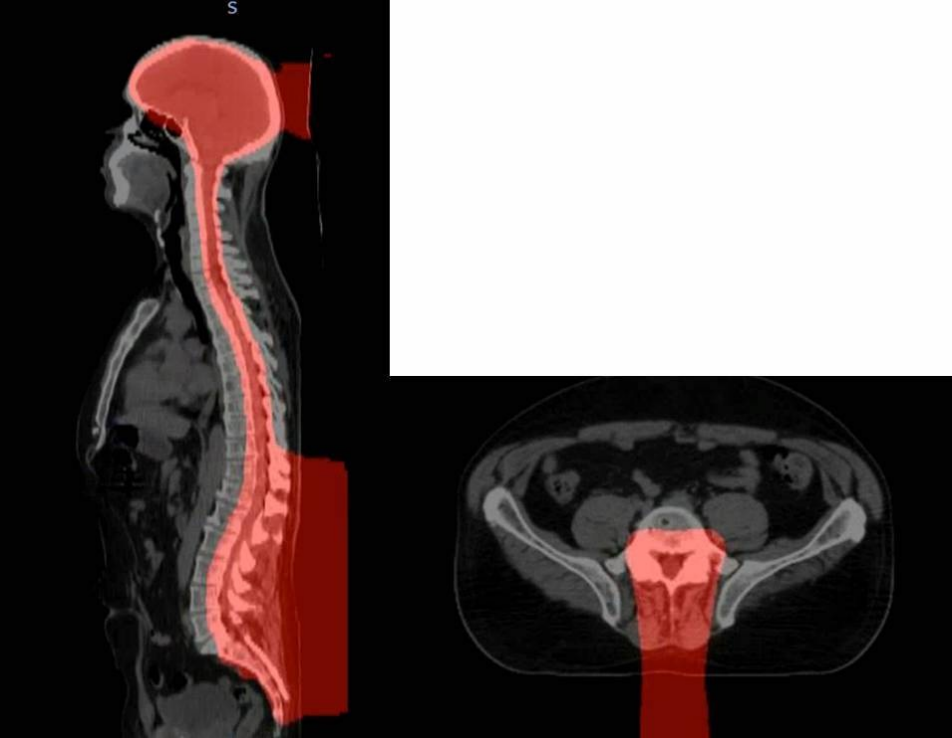
Bone marrow preserving proton therapy craniospinal irradiation. The finite distal range of posterior proton therapy beams were placed in the middle of the vertebral bodies of the spine, thereby limiting the radiation dose to the bone marrow that was already treatment sensitive due to multiple prior chemotherapy regimens and autologous stem cell transplant. The red colored space represents the 20 cobalt Gray equivalent (CGyE) isodose cloud.

Given his history of stem cell transplant, the proton beam therapy with bone marrow-sparing was recommended to minimize the risk of hematologic toxicity (Figure 2). To achieve bone marrow-sparing, the posterior beams were utilized with the finite distal range of the proton beams placed in the dorsal vertebral body just anterior to the CSF space. A dose of 20.06 cobalt Gray equivalents (CGE) at 2 CGE per fraction was delivered to the entire neuraxis followed by a boost to areas of bulky disease in the brain and cauda equina to a cumulative dose of 30.04 CGE.

**Figure 3:**
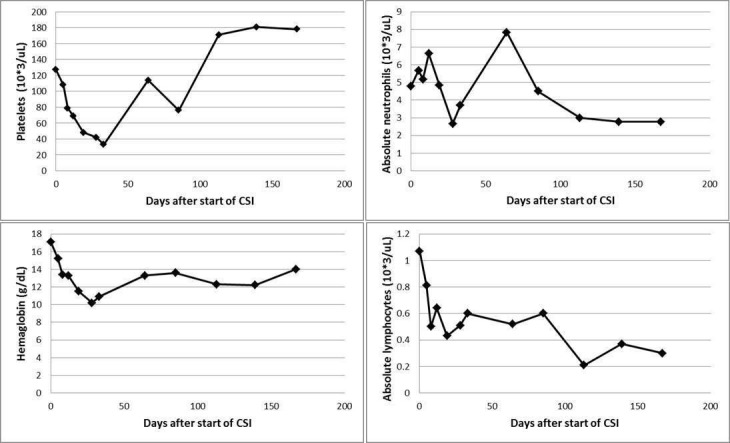
Peripheral blood counts at the baseline, during and after craniospinal irradiation.

The radiation therapy was fairly well tolerated with complete resolution of the neurologic deficits. His peripheral blood counts were monitored bi-weekly during the treatment and for several weeks after the treatment (Figure 3). He required only one unit platelet transfusion for radiotherapy-related thrombocytopenia, one week after completion of the treatment. Otherwise, acute toxicity was limited to mild scalp erythema.

After completion of craniospinal irradiation, he resumed systemic therapy with pomalidomide and dexamethasone with close neuro-oncologic surveillance. At his last follow-up visit, approximately nine months after completion of proton therapy, follow-up brain MRI with contrast showed durable near complete radiographic response (Figure 1), and he was doing well with no evidence of relapse. The left frontal intracranial lesion continued to decrease in size on systemic therapy.

## Discussion

The CNS involvement is a rare but increasingly recognized complication of the multiple myeloma. Awareness of this entity is important given improved survival in the myeloma patients in recent decades, particularly in younger age groups. High-risk cytogenetics, plasmablastic morphology, and the extramedullary myeloma involvement have been associated with increased risk of CNS MM [1]. The CNS disease typically develops in advanced stage disease after multiple prior treatments. The presenting symptoms may include headaches, focal neurologic symptoms (in particular, cranial neuropathies), altered mental status or spinal radiculopathies [2]. Lumbar puncture with the examination of the CSF and MRI of the complete neuraxis are essential for accurate diagnosis. Leptomeningeal enhancement is a common finding on MRI, while parenchymal brain lesions are relatively uncommon [2].

Prognosis after the development of CNS myeloma is dismal, with survival typically in the order of one to six months [3-5]. There is no standard therapeutic approach for CNS MM, however, previous reports have described transient responses to chemotherapy, radiotherapy or a combination of both. In a case-series of 37 multiple myeloma patients with CNS involvement, prolonged survival was associated with multi-modal therapy including intrathecal chemotherapy, radiotherapy and immunomodulatory agents [2].

Although novel systemic agents have led to improved outcomes for multiple myeloma patients, the majority of these agents has limited penetration of the blood-brain barrier and therefore, are relatively ineffective in the treatment of CNS myeloma [3]. In a retrospective analysis by the Greek Myeloma Study Group, the receipt of novel agents such as thalidomide, Bortezomib, and lenalidomide was not associated with improved outcomes after the development of CNS myeloma [3]. However, based on a systematic review including 109 cases of CNS MM, Nieuwenhuizen, et al. found that CSI was associated with prolonged survival compared to no CSI (median survival of three months vs 0.81 months), supporting the role of radiation therapy in this setting [5].

Hematologic toxicity is a potential concern when considering CSI in multiple myeloma due to prior exposure to several myelosuppressive chemotherapy agents and, in many cases, the use of autologous stem cell transplant. For example, grade three- four hematologic toxicity was seen in 33% of the patients with primary CNS tumors treated with photon CSI at the Royal Marsden Hospital [6]. Prior receipt of chemotherapy has been identified as a risk factor for hematologic toxicity [7]. In one study, severe leukopenia/thrombocytopenia requiring a treatment break was seen in 47% of the patients who had received chemotherapy prior to CSI compared to only 5% in those who had not (p <0.01) [7]. In addition to prior chemotherapy, the “low dose bath” associated with photon-based CSI may be an important contributor to hematologic toxicity. This was suggested in a study of photon CSI delivered with helical tomotherapy or three-dimensional conformal radiation therapy, in which there was a correlation between the volume of the red bone marrow exposed to doses between 2-6 Gy and the severity of hematologic toxicity [8].

The proton beam CSI may reduce hematologic toxicity compared to photon CSI due to superior sparing of bone marrow in the vertebral column. In a study of adults with medulloblastoma, the proton beam CSI was associated with reduced rates of grade one or higher anemia compared to photon CSI (17% vs 48%, p=0.04) [9]. The relatively low hematologic toxicity profile with proton beam CSI was confirmed in a larger analysis of the adult patients with a variety of malignancies treated with proton beam CSI at M.D. Anderson Cancer Center, in which only 4/50 (8%) patients developed severe cytopenias despite the majority receiving chemotherapy [10].

Although there are no standard indications for proton beam CSI, these studies suggest that this modality might be considered in special circumstances to reduce hematologic toxicity, such as in the patients with prior chemotherapy or stem cell transplant. Furthermore, the proton CSI is widely utilized in pediatric medulloblastoma both for its decreased long-term extra-CNS toxicity as well as its bone marrow sparing.

## Conclusions

In conclusion, the myeloma involvement of the CNS is rare and associated with poor prognosis. The radiotherapy in the form of CSI is an important therapeutic tool in the treatment of CNS MM and should be considered early in the disease course. Bone marrow-sparing CSI using proton therapy is a useful technique to limit hematologic toxicity, control and reverse neurologic sequelae from disease progression, and prolong survival, as illustrated in this case report.
